# A Retrospective Survey of Research Design and Statistical Analyses in Selected Chinese Medical Journals in 1998 and 2008

**DOI:** 10.1371/journal.pone.0010822

**Published:** 2010-05-25

**Authors:** Zhichao Jin, Danghui Yu, Luoman Zhang, Hong Meng, Jian Lu, Qingbin Gao, Yang Cao, Xiuqiang Ma, Cheng Wu, Qian He, Rui Wang, Jia He

**Affiliations:** 1 Department of Health Statistics, Second Military Medical University, Shanghai, China; 2 Academic Journal of Second Military Medical University, Shanghai, China; Copenhagen University Hospital, Denmark

## Abstract

**Background:**

High quality clinical research not only requires advanced professional knowledge, but also needs sound study design and correct statistical analyses. The number of clinical research articles published in Chinese medical journals has increased immensely in the past decade, but study design quality and statistical analyses have remained suboptimal. The aim of this investigation was to gather evidence on the quality of study design and statistical analyses in clinical researches conducted in China for the first decade of the new millennium.

**Methodology/Principal Findings:**

Ten (10) leading Chinese medical journals were selected and all original articles published in 1998 (N = 1,335) and 2008 (N = 1,578) were thoroughly categorized and reviewed. A well-defined and validated checklist on study design, statistical analyses, results presentation, and interpretation was used for review and evaluation. Main outcomes were the frequencies of different types of study design, error/defect proportion in design and statistical analyses, and implementation of CONSORT in randomized clinical trials. From 1998 to 2008: The error/defect proportion in statistical analyses decreased significantly (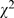 = 12.03, p<0.001), 59.8% (545/1,335) in 1998 compared to 52.2% (664/1,578) in 2008. The overall error/defect proportion of study design also decreased (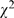 = 21.22, p<0.001), 50.9% (680/1,335) compared to 42.40% (669/1,578). In 2008, design with randomized clinical trials remained low in single digit (3.8%, 60/1,578) with two-third showed poor results reporting (defects in 44 papers, 73.3%). Nearly half of the published studies were retrospective in nature, 49.3% (658/1,335) in 1998 compared to 48.2% (761/1,578) in 2008. Decreases in defect proportions were observed in both results presentation (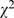 = 93.26, p<0.001), 92.7% (945/1,019) compared to 78.2% (1023/1,309) and interpretation (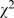 = 27.26, p<0.001), 9.7% (99/1,019) compared to 4.3% (56/1,309), some serious ones persisted.

**Conclusions/Significance:**

Chinese medical research seems to have made significant progress regarding statistical analyses, but there remains ample room for improvement regarding study designs. Retrospective clinical studies are the most often used design, whereas randomized clinical trials are rare and often show methodological weaknesses. Urgent implementation of the CONSORT statement is imperative.

## Introduction

High quality clinical research not only requires advanced professional knowledge, but also needs sound study design and correct statistical analyses. Yates and Healy [1] once stated, “It is depressing to find how much good biological work is in danger of being wasted through incompetent and misleading analyses of numerical results.”

Since the early 1970s, Altman et al. [2]–[6] have studied the errors/defects in all aspects of study design and statistical analyses in medical journals. Consequently, a series of report guidelines were proposed, including CONSORT and TREND statements [7]–[10]. These guidelines have greatly improved the quality of publications in clinical research worldwide. Nonetheless, the influence of these guidelines on the Chinese scientists remains unclear. Though there were a few articles that had addressed the quality of statistical application. However, most of them had focused on qualitative analyses instead of quantitative one, and were limited to listing the errors/defects in study design and statistical analyses inclusive. Wang and Zhang reviewed the published articles in five Chinese medical journals in 1995 [11]. They performed a cross-sectional analysis on the types of study design, statistical analyses, and made a quantitative analyses of the errors in statistical methods.

The number of clinical research papers published by Chinese scientists has greatly increased in the past decade. This research intends to provide an updated perspective on clinical research in China. It compared the errors/defects in study design and statistical analyses published in 1998 and 2008 in 10 leading Chinese medical journals. A total of 2,913 articles from 228 issues in 1998 (N = 1335) and 2008 (N = 1578) were reviewed. We evaluated the study design, statistical analyses, and results presentation and interpretation. We also examined randomized clinical trials according to the CONSORT statement. In addition, the current status and trends of study design and statistical analyses were discussed and remedies for improvement were proposed.

## Methods

### Journals

There are nearly 1,100 biomedical journals in China and less than 100 being indexed by Medline. For this research we selected 10 leading medical journals published in Chinese, sponsored by Chinese Medical Association (CMA) and indexed by Medline. The 10 journals are: *Chinese Journal of Internal Medicine*, *Chinese Journal of Surgery*, *Chinese Journal of Pediatrics*, *Chinese Journal of Obstetrics and Gynecology*, *Chinese Journal of Ophthalmology*, *Chinese Journal of Hematology*, *Chinese Journal of Stomatology*, *Chinese Journal of Cardiology*, *Chinese Journal of Oncology*, *and Chinese Journal of Tuberculosis and Respiratory Diseases* (see Table S1).

All articles published in these journals were peer reviewed. We examined all the original articles published in 1998 and 2008. The review contents included the types of study design, frequencies of various statistical methods, errors/defects in study design and statistical analyses, and implementation of CONSORT in randomized clinical trials.

### Quality control

Rigorous quality control was implemented throughout this investigation. As to the definitions of different types of study designs, we mainly followed the definitions and standards established by The Centre for Evidence-Based Medicine, Oxford UK [12]. Meanwhile, considering the situations in China, two new types, namely case study and case series study, were also included. The standards of quality for different study designs were derived from the corresponding statements, such as CONSORT, STROBE, STARD and TREND statement [7]–[10]. Statistical methods were categorized by a modified method used by Emerson [13]. We counted all the statistical methods used in an article, but if a method was used repeatedly in that article, it was only counted once. After three rounds of pre-survey and five rounds of Delphi method and team discussion, a well-defined checklist with specifications was established (see Appendix S1). Regarding randomized clinical trials, most Chinese medical journals have not yet followed the CONSORT statement. In fact, there are only 4 journals have endorsed the CONSORT statement to date (i.e., *Chinese Journal of Evidence-based Medicine*, *Chinese Medical Journal*, *Chinese Medicine*, *Journal of Chinese Integrative Medicine*). We examined the randomized clinical trials by several main issues of the CONSORT statement related to statistics. Twenty-one researchers participated in this study: 5 professors, 7 lecturers, 4 assistants and 5 postgraduates. All of them have received formal training in health statistics and some of them have long-term teaching and research experience. Each article was reviewed by 3 researchers independently, including a professor, a lecturer and an assistant or postgraduate. Discrepancies were solved by team discussion.

### Data analysis

Epidata3.1 was used for double data entry and management. SAS9.1.3 was used for analysis, as appropriate.

## Results

### Situation of study design

As shown in Table S1 and S2 from 1998 to 2008, both the numbers of issues and articles in the 10 journals were increased (issues: 96 to 132; articles: 1335 to 1578). The basic science studies increased significantly (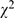 = 10.61, p = 0.001), 24.3% (324/1,335) in 1998 compared to 29.7% (468/1,678) in 2008. Surprisingly, the number of clinical trials remained low in single digit (randomized clinical trials: 4.9% (66/1335) compared to 3.8% (60/1,578); non- randomized clinical trials: 6.7% (90/1,335) compared to 3.9% (61/1,578)). The majority of the published studies remained retrospective, 49.3% (658/1,335) compared to 48.2% (761/1,578). However, the overall error/defect proportion of study design was decreased (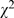 = 21.22, p<0.001), 50.9% (680/1,335) compared to 42.4% (669/1,578). In general, randomized clinical trials, non- randomized clinical trials, cohort study and case-control study tended to use statistical analyses more frequently.

No randomized clinical trials or non- randomized clinical trials were found being registered on the domestic or international clinical trial registries. Based on the checklist derived from CONSORT, the error/defect proportion of randomized clinical trials had dropped markedly (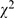 = 6.74, P = 0.009), 90.9% (60/66) in 1998 compared to 73.3% (44/60) in 2008, despite there were small number of randomized clinical trials. Omission of sample size estimation, failure to use (or report) randomization, failure to use (or report) blinding, and unclear primary outcome measures were the most common errors/defects in randomized clinical trials design (Table S3). A notable improvement in error/defect proportion was observed in sample size estimation (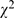 = 7.68, p = 0.006), 84.9% (56/66) compared to 63.3% (38/60); However, more than one-half of the articles still failed to meet this vital requirement in conducting quality clinical trials. Cases were also improved for randomization and blinding, but most articles only mentioned the use without describing how the randomization and blinding were done.

### Situation of statistical analyses

As shown in Table S4, articles using statistical methods had increased markedly in 2008 (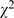 = 35.94, p<0.001), 68.3% (912/1,335) in 1998 compared to 78.1% (1,233/1,578) in 2008. In 1998, 31.7% (423/1,335) articles had no statistical analyses, in which 8.0% (107/1,335) needed statistical analyses but omitted. In 2008, 21.9% (345/1,578) articles had no statistical analyses, in which 4.8% (76/1,578) needed but had statistical analyses omitted. The most used statistical methods remained the simple tests (i.e., t-tests, contingency table and ANOVA). Some more sophisticated statistical methods, such as repeated-measures analysis, logistic regression and survival analysis emerged in 2008. The error/defect proportion of statistical analyses had decreased (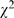 = 12.03, p<0.001), 59.8% (545/1,335) compared to 52.2% (664/1,578). Statistical methods that had been misused frequently were also observed in these simple tests. Compared with 1998, the error proportions of t-test and contingency table were also noticeably decreased (t-test: 62.0% (305/492) compared to 44.4% (253/570), 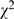 = 32.83, p<0.001; contingency table: 48.3% (154/319) compared to 32.3% (169/523), 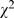 = 21.35, p<0.001). The most common mistakes for these three methods were using multiple t-tests for multiple group comparisons, absence of significant level adjustment for multiple comparisons in contingency table, ignoring or misusing the method of multiple pair-wise comparisons in ANOVA.

### Results presentation and interpretation

Beside study design and statistical analyses, presentation and interpretation of results also improved, but serious errors/defects persisted (Table S5). Inappropriate presentation of statistical results was the most common defect seen. Using arbitrary p thresholds instead of reporting exact p values, reporting p value without showing test statistics, and insufficient (or inappropriate) description of methods were common. The overall proportion of inappropriate presentation of results decreased significantly (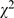 = 93.26, p<0.001), 92.7% (945/1,019) in 1998 compared to 78.2% (1,023/1,309) in 2008; In 2008, using arbitrary p thresholds instead of reporting exact p values had decreased to 61.7% (807/1,309, 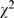 = 133.62, p<0.001); reporting p value without test statistics had dropped to 57.6% (754/1,309, 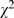 = 74.44, p<0.001); inappropriate description of statistical methods was also down to 38.7% (506/1,309, 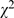 = 45.69, p<0.001). Also, the proportion of inappropriate interpretation of results was decreased (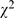 = 27.26, p<0.001), 9.7% (99/1,019) compared to 4.3% (56/1,309). The most common error in interpreting the results was the misconception of p value, that is when p<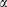, the smaller the p-value is, the greater the difference between groups is. This error was 3.3% (34/1,019) in 1998 compared to 0.2% (2/1,309) in 2008.

## Discussion

Randomized clinical trial is considered as the “gold standard” for clinical trials. To our surprise in 2008, after 10 years' steady progress, randomized clinical trials published in Chinese medical journals remained low (less than 5%). Along the same line, the quality of study design and statistical analyses of the randomized clinical trials merely improved marginally. There might be multiple reasons. First, Chinese clinicians, in general, do not have concrete training in study design. West China University of Medical Sciences and Shanghai Medical University were the sole pioneers to have offered such courses since 1983. Second, some better quality research papers have been submitted and published in English in international journals.

In 2008, Xu *et al* assessed the randomized clinical trials published in Chinese medical journal and found that only 22 articles (15.5%) reached a high quality grade (≥3 points) [14]. Compared with a failure proportion of 73.2% in reporting randomization in the findings of Xu et al., we found a lower failure proportion of 30.3%. It might due to the fact that we selected the randomized clinical trials with rigorous criteria. We categorized them into non- randomized clinical trials even they had “randomized clinical trials” in their titles, if they were indeed non-randomized trials. Wu et al [15] interviewed the authors of 2,235 randomized clinical trials, and found that only 207 studies could be considered to have performed real randomization. In 2007, China launched a system named Chinese Clinical Trial Registration and Publication Collaboration (ChiCTRPC) [16]. Since then Forty-eight journals have joined, not including the 10 leading journals we had selected. Li et al. [16] declared that the member of ChiCTRPC will be given priority in publishing clinical trials with unique registration number than those non-members. We strongly endorse the requirement for full implementation of the clinical trial registration system to promote the quality of randomized clinical trials in China.

We found that there was little change in the types of study design in 2008 compared to 1998. Retrospective studies remain ‘main stream’. Prospective clinical research, including randomized clinical trials and non- randomized clinical trials, only accounted for 8.1% in 2008. In 1991, McDermott *et al* found that 35.0% papers published in *JAMA*, *The Lancet* and *New England Journal of Medicine* was clinical trials [17]. Clinicians should take the advantage of China's large population, rich case source, broad disease spectrum, and low cost to conduct high quality randomized clinical trials. Health policy-makers should proactively encourage clinical research via randomized clinical trials with relevant guidance to the researchers to pay more attention to quality than quantity of their publications.

As we reported previously, the quality of statistical analyses used in Chinese medical research has been greatly improved. However, much more work still needs to be done as point out by He et al. [18]. In the present study we found that articles using statistical methods increased noticeably and the use of sophisticated statistical methods also have emerged. This was similar to the findings of Horton and Switzer in 2005 [19], in which they reported that the use of survival analysis, multiple regression analysis, and repeated measure analysis had greatly increased. We also noticed an impressive increase in the use of rank based nonparametric test in 2008, indicating that more attention had been paid to the precondition of parametric test. The progress is mainly attributable to the emphasis of statistical education among medical postgraduates in China. Serious problems remained nonetheless. Simple methods like t-tests, contingency tables, and ANOVA are likely to be used incorrectly. As for the defects in statistical result presentation, even prestigious journals like *Nature* and *BMJ* had a defect proportion of 38.0% and 25.0%, respectively [20]–[21]. In this study, we found the most common defects in presentation of results were using arbitrary p thresholds instead of reporting the precise p values and reporting a p value without showing the test statistics. Precise p value and test statistic are better be given at the same time [6], [20]. For interpretation of results, the main problem was that the authors considered that there was a trend of difference between groups when p>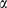 without giving a thought regarding how large the p value was. Pocock and Ware [22] suggested that “trend” in this context should be avoided because it implied special pleading even the evidence was slim. Medical colleges should emphasize teaching of the basic statistical concepts and strengthen statistical thinking among medical students. In hospitals, continuous education on biostatistics should be encouraged among clinicians. For journal editorial board, qualified statistician should be involved with statue strengthened.

The journals selected in the present study covered the important clinical fields and represented the top academic level of China. One thing we should point out is that some excellent Chinese research papers are published in high-level international journals elsewhere outside China. Unfortunately, these articles were not included. Another limitation is that, due to inadequate background description by authors or the limited clinical knowledge of our reviewers, we do not know whether a treatment is based on the design of the researchers or a conventional therapy, and this may cause the discrepancies between intervention and exposure among reviewers.

In summary, this study indicates that Chinese medical research seems to have made significant progress regarding statistical analyses, but there remains ample room for improvement regarding study designs. Retrospective clinical studies are the most often used design, whereas randomized clinical trials are rare and often show methodological weaknesses. Absence of sample size estimation and power consideration as well as failure in (or reporting) randomization is common. Full implementation of the CONSORT statement and registration system for clinical trial is an urgent task. Compared with the clinical researches in the developed countries, clinical research in China still has ample rooms for improvement, not only in clinical professional knowledge, but also in study design and statistical analyses. Urgent implementation of the CONSORT statement is imperative. In addition, to improve the situation a system project which requires close collaboration among the medical colleges, clinical researchers, statisticians, journal editors and reviewers, as well as the health policy-makers would also be greatly beneficial.

## Supporting Information

Appendix S1 Checklist for study design and statistical analysis in Chinese medical journals. This checklist was used to record all the related items through the paper review.
(0.08 MB PDF)

Table S1 General information of the 10 selected leading Chinese medical journals in 1998 and 2008. Both the numbers of issues and articles in the 10 journals were increased.
(0.04 MB DOC)

Table S2 Study designs and articles used statistical analyses. The majority of the published studies remained retrospective. However, the overall error/defect proportion of study design was decreased. Randomized clinical trials, non- randomized clinical trials, cohort study and case-control study tended to use statistical analyses more frequently.
(0.05 MB DOC)

Table S3 Error/Defects in randomized clinical trial design. The report of randomized clinical trials still poor. Omission of sample size estimation, failure to use (or report) randomization, failure to use (or report) blinding, and unclear primary outcome measures were the most common errors/defects in randomized clinical trials design.
(0.04 MB DOC)

Table S4 Statistical methods. Articles using statistical methods had increased markedly in 2008. The error/defect proportion of statistical analyses had decreased significantly.
(0.05 MB DOC)

Table S5 Inappropriate presentation a/o interpretation of results. Presentation and interpretation of results have been improved, but serious errors/defects persisted. Inappropriate presentation of statistical results was the most common defect seen.
(0.04 MB DOC)
