# Supplementary material for: A Retrospective Survey of Research Design and Statistical Analyses in Selected Chinese Medical Journals in 1998 and 2008
Source: PLoS One. 2010 May 25;5(5):e10822. doi: 10.1371/journal.pone.0010822 (PMC2876024; doi:10.1371/journal.pone.0010822)
Supplement: Appendix S1 — Checklist for study design and statistical analysis in Chinese medical journals. This checklist was used to record all the related items through the paper review. (0.08 MB PDF) [file pone.0010822.s001.pdf]

Checklist for study design and statistical analysis in Chinese medical Journals

Table1 Errors/defects in study design of observational research

Study Design	Applied	Misused	Types of error
Case Study or Case Series Study	☐YES<br>☐NO	☐YES<br>☐NO	
Cross-Sectional Study	☐YES<br>☐NO	☐YES<br>☐NO	1 ☐ No sampling when needed<br>2 ☐ Inappropriate sampling method or procedure<br>3 ☐ No details of sampling procedure<br>4 ☐ No sample size estimating step<br>5 ☐ No analysis for non-response samples<br>Others，please specify：
Cohort Study	☐YES<br>☐NO	☐YES<br>☐NO	1 ☐ No sampling when needed<br>2 ☐ Inappropriate sampling method or procedure<br>3 ☐ No details of sampling procedure<br>4 ☐ No sample size estimating step<br>5 ☐ No inclusion and exclusion criteria<br>6 ☐ Inappropriate non-exposed group<br>7 ☐ No analysis for withdrawals<br>Others，please specify：
Case-Control Study	☐YES<br>☐NO	☐YES<br>☐NO	1 ☐ No sampling when needed<br>2 ☐ Inappropriate sampling method or procedure<br>3 ☐ No details of sampling procedure<br>4 ☐ No sample size estimating step<br>5 ☐ No inclusion and exclusion criteria<br>6 ☐ Inappropriate control group<br>Others，please specify：
Diagnostic Test	☐YES<br>☐NO	☐YES<br>☐NO	1 ☐ No sample size estimating step<br>2 ☐ No control group or inappropriate control group<br>Others，please specify：

Table 2 Errors/defects in study design of intervention research

Study design	Applied	Misused	Types of error
RCT	☐YES<br>☐NO	☐YES<br>☐NO	1 ☐ Unclear study aim and hypothesis<br>2 ☐ Unclear primary outcome measures<br>3 ☐ No sample size estimating step<br>4 ☐ No inclusion and exclusion criteria<br>5 ☐ No statement of intervention for each group or unclear<br>6 ☐ Failure to use or report randomization<br>7 ☐ No report of blindness when needed<br>8 ☐ No analysis for withdrawals<br>Others，please specify：
	Does RCT register on clinical trail registration platform? 0 ☐ NO， 1 ☐ YES		
Non-RCT	☐YES<br>☐NO	☐YES<br>☐NO	1 ☐ Unclear study aim and hypothesis<br>2 ☐ Unclear primary outcome measures<br>3 ☐ No inclusion and exclusion criteria<br>4 ☐ No sample size estimating step when needed<br>5 ☐ Failure to use or report randomization if possible<br>6 ☐ Failure to use or report blinding if possible<br>7 ☐ No statement of intervention for each group or unclear<br>8 ☐ No analysis for withdrawals<br>Others，please specify：
Basic science study*	☐YES<br>☐NO	☐YES<br>☐NO	1 ☐ No sample size estimating step<br>2 ☐ Failure to report the test power<br>3 ☐ No statement of intervention for each group or unclear<br>4 ☐ Failure to use or report randomization<br>5 ☐ No control group<br>6 ☐ Use of inappropriate control group<br>Others，please specify：

*Basic science study includes preclinical animal and laboratory in vivo and in vitro study.

Does the research need statistical analysis? 0☐NO 1☐YES, if “YES”， fail to use? 0☐NO 1☐YES

Table 3 Statistical content and errors/defects in original articles

Statistical methods	Applied	Misused	Type of errors
Statistical description	☐YES<br>☐NO	☐YES<br>☐NO	
t-test	☐YES<br>☐NO	☐YES<br>☐NO	1 ☐ Using multiple t-test for multiple group comparison<br>2 ☐ Using paired t-test for unpaired data or vice versa<br>3 ☐ Using t-test under nonparametric setting<br>4 ☐ Using t-test without considering the baseline<br>Others，please specify：
Contingency tables	☐YES<br>☐NO	☐YES<br>☐NO	1 ☐ No continuity correction or Fisher exact test if needed<br>2 ☐ No significant level adjustment for multiple comparison<br>3 ☐ Misusing Chi-square test for paired fourfold table<br>4 ☐ Using Chi-square test for ranked data<br>5 ☐ Ignorance of stratification factors<br>6 ☐ Using p value of Chi-square test to describe the correlation of two variables instead of contingency coefficient<br>Others，please specify：
Rank transformation nonparametric test	☐YES<br>☐NO	☐YES<br>☐NO	1 ☐ Using multiple pair-wise comparison for multiple group comparison<br>2 ☐ Using wrong type of rank sum test for different study types<br>Others，please specify：
ANOVA	☐YES<br>☐NO	☐YES<br>☐NO	1 ☐ Using completely random ANOVA to analyze multi-factor ANOVA<br>2 ☐ Ignoring the setting of ANOVA for completely random design data<br>3 ☐ No multiple pair-wise comparison of ANOVA when needed<br>4 ☐ Misusing the method of multiple pair-wise comparison of ANOVA<br>5 ☐ Using ANOVA to analyze repeated-measures data<br>Others，please specify：
Repeated-measures analysis	☐YES<br>☐NO	☐YES<br>☐NO	
Analysis of covariance	☐YES<br>☐NO	☐YES<br>☐NO	
Pearson’s correlation	☐YES<br>☐NO	☐YES<br>☐NO	

Simple linear regression	☐YES ☐NO	☐YES ☐NO	
Nonlinear regression	☐YES ☐NO	☐YES ☐NO	
Nonparametric correlation	☐YES ☐NO	☐YES ☐NO	
Multiple linear regression	☐YES ☐NO	☐YES ☐NO	
Logistic regression	☐YES ☐NO	☐YES ☐NO	
Survival analysis	☐YES ☐NO	☐YES ☐NO	
Clustering analysis	☐YES ☐NO	☐YES ☐NO	
Discriminant analysis	☐YES ☐NO	☐YES ☐NO	
Principle component analysis and factor analysis	☐YES ☐NO	☐YES ☐NO	
Canonical correlation analysis	☐YES ☐NO	☐YES ☐NO	
Meta analysis	☐YES ☐NO	☐YES ☐NO	
ROC	☐YES ☐NO	☐YES ☐NO	
Reliability and validity analysis	☐YES ☐NO	☐YES ☐NO	
Others，please specify：			

Table 4 Inappropriate presentation a/o interpretation of results

Result presentation and interpretation	YES/NO
**Types of inappropriate result presentation**	
Unspecified statistical methods	☐YES ☐NO
Insufficient or inappropriate description of methods	☐YES ☐NO
Reporting *p* value without test statistics	☐YES ☐NO
p values without confidence intervals	☐YES ☐NO
Using mean±SD to describe non-normal data	☐YES ☐NO
Using proportion instead of rate	☐YES ☐NO
Using arbitrary *p* thresholds (like $p<0.01$) instead of reporting exact *p* values	☐YES ☐NO
Incorrect use of table	☐YES ☐NO
Incorrect use of chart and figure	☐YES ☐NO
Ignoring baselines of two groups	☐YES ☐NO
Others，please specify：	
**Types of inappropriate result interpretation**	
$p<\alpha$， the smaller the *P* value, the greater the difference between groups	☐YES ☐NO
$p>\alpha$， reporting no difference between groups	☐YES ☐NO
$p>\alpha$， reporting difference between groups	☐YES ☐NO
Others，please specify：	
