# Supplementary material for: A Retrospective Survey of Research Design and Statistical Analyses in Selected Chinese Medical Journals in 1998 and 2008
Source: PLoS One. 2010 May 25;5(5):e10822. doi: 10.1371/journal.pone.0010822 (PMC2876024; doi:10.1371/journal.pone.0010822)
Supplement: Table S1 — General information of the 10 selected leading Chinese medical journals in 1998 and 2008. Both the numbers of issues and articles in the 10 journals were increased. (0.04 MB DOC) [file pone.0010822.s002.doc]

| **Table S1. General information of the 10 selected leading Chinese medical journals** | | | | | |
| --- | --- | --- | --- | --- | --- |
| Journals | 1998 | |  | 2008 | |
|  | # Issues | # Articles |  | # Issues | # Articles |
| Chinese Journal of Internal Medicine | 12 | 122 |  | 12 | 128 |
| Chinese Journal of Surgery | 12 | 207 |  | 24 | 343 |
| Chinese Journal of Pediatrics | 12 | 140 |  | 12 | 124 |
| Chinese Journal of Obstetrics and Gynecology | 12 | 136 |  | 12 | 144 |
| Chinese Journal of Ophthalmology | 6 | 114 |  | 12 | 147 |
| Chinese Journal of Hematology | 12 | 105 |  | 12 | 127 |
| Chinese Journal of Stomatology | 6 | 108 |  | 12 | 128 |
| Chinese Journal of Cardiology | 6 | 115 |  | 12 | 198 |
| Chinese Journal of Oncology | 6 | 141 |  | 12 | 135 |
| Chinese Journal of Tuberculosis and Respiratory Diseases | 12 | 147 |  | 12 | 104 |
| Total | 96 | 1335 |  | 132 | 1578 |
