# Supplementary material for: A Retrospective Survey of Research Design and Statistical Analyses in Selected Chinese Medical Journals in 1998 and 2008
Source: PLoS One. 2010 May 25;5(5):e10822. doi: 10.1371/journal.pone.0010822 (PMC2876024; doi:10.1371/journal.pone.0010822)
Supplement: Table S2 — Study designs and articles used statistical analyses. The majority of the published studies remained retrospective. However, the overall error/defect proportion of study design was decreased. Randomized clinical trials, non- randomized clinical trials, cohort study and case-control study tended to use statistical analyses more frequently. (0.05 MB DOC) [file pone.0010822.s003.doc]

| **Table S2. Study designs and articles used statistical analyses** | | | | | | | |  |
| --- | --- | --- | --- | --- | --- | --- | --- | --- |
| Study Design | 1998 | | |  | 2008 | | |  |
|  | # Articles | # Articles used statistical analyses  n (%)* | # Articles had design defects  n (%) |  | # Articles | # Articles used statistical analyses  n (%)* | # Articles had design defects  n (%) | |
| Systematic Review | 0 | 0 | 0 |  | 6  (0.38%) | 6  (100%) | 0 |  |
| Randomized clinical trial | 66  (4.9%) | 64 (97.0%) | 60  (90.91%) |  | 60 (3.80%) | 56 (93.3%) | 45  (75.00%) |  |
| Non- randomized clinical trial | 90  (6.7%) | 82 (91.1%) | 67  (74.44%) |  | 61 (3.9%) | 58 (95.1%) | 41  (67.21%) |  |
| Cohort Study | 59  (4.4%) | 47 (79.7%) | 39  (66.10%) |  | 87 (5.5%) | 80 (92.0%) | 41  (47.13%) |  |
| Case-Control Study | 275 (20.6%) | 254 (92.4%) | 194  (70.55%) |  | 284 (18.0%) | 276 (97.2%) | 189  (66.55%) |  |
| Cross-Sectional Study | 75  (5.6%) | 56 (74.7%) | 43  (57.33%) |  | 59 (3.7%) | 52 (88.1%) | 38  (64.41) |  |
| Case Study or Case Series Study | 383 (28.7%) | 122 (31.9%) | 0 |  | 477 (30.2%) | 233 (48.9%) | 0 |  |
| Diagnostic Test | 63  (4.7%) | 47 (74.6%) | 49  (77.78%) |  | 76 (4.8%) | 63 (82.9%) | 46  (60.53%) |  |
| Basic science study** | 324 (24.3%) | 240 (74.1%) | 228  (70.37%) |  | 468 (29.7%) | 409 (87.4%) | 269  (57.48%) |  |
| Total | 1335 (100.0%) | 912 (68.3%) | 680  (50.94%) |  | 1578 (100.0%) | 1233 (78.1%) | 669  (42.40%) |  |

*No. of articles used statistical analyses n (%): n is the number of articles using statistical methods, percentage=n/the number of articles with the certain study design×100%.

**Basic science study includes preclinical animal and laboratory in vivo and in vitro study.
