# Supplementary material for: A Retrospective Survey of Research Design and Statistical Analyses in Selected Chinese Medical Journals in 1998 and 2008
Source: PLoS One. 2010 May 25;5(5):e10822. doi: 10.1371/journal.pone.0010822 (PMC2876024; doi:10.1371/journal.pone.0010822)
Supplement: Table S3 — Error/Defects in randomized clinical trial design. The report of randomized clinical trials still poor. Omission of sample size estimation, failure to use (or report) randomization, failure to use (or report) blinding, and unclear primary outcome measures were the most common errors/defects in randomized clinical trials design. (0.04 MB DOC) [file pone.0010822.s004.doc]

| **Table S3. Error/Defects in randomized clinical trial design** | | |
| --- | --- | --- |
| Error/Defect in randomized clinical trial | 1998  # articles  n (%)  (N=66) | 2008  # articles  n (%)  (N=60) |
| Unclear study objective and hypothesis | 15 (22.7%) | 16 (26.7%) |
| Unclear primary outcome measures | 18 (27.3%) | 15 (25.0%) |
| No sample size estimation | 56 (84.9%) | 38 (63.3%) |
| No inclusion and exclusion criteria | 13 (19.7%) | 9(15.0%) |
| No (or unclear) statement of intervention for each group | 5 (7.6%) | 0 (0.0%) |
| Failure to use (or report) randomization | 30 (45.5%) | 20 (33.3%) |
| No report of blinding when needed | 21 (31.8%) | 15 (25.0%) |
| No analysis on dropouts/withdrawals | 17 (25.8%) | 12 (20.0%) |
| Total | 60 (90.9%) | 44 (73.3%) |

N= total articles with randomized clinical trial design
