# Supplementary material for: A Retrospective Survey of Research Design and Statistical Analyses in Selected Chinese Medical Journals in 1998 and 2008
Source: PLoS One. 2010 May 25;5(5):e10822. doi: 10.1371/journal.pone.0010822 (PMC2876024; doi:10.1371/journal.pone.0010822)
Supplement: Table S4 — Statistical methods. Articles using statistical methods had increased markedly in 2008. The error/defect proportion of statistical analyses had decreased significantly. (0.05 MB DOC) [file pone.0010822.s005.doc]

| **Table S4. Statistical methods** | | | | | |
| --- | --- | --- | --- | --- | --- |
| Statistical methods | 1998 (N=1335) | |  | 2008 (N=1578) | |
|  | All articles  n (%) | Incorrect use  n (%) |  | All articles  n (%) | Incorrect use  n (%) |
| No statistical methods or description* | 423 (31.7%) | - |  | 345(21.9%) | - |
| Articles using statistical methods | 912 (68.3%) | 545 (59.8%) |  | 1233 (78.1%) | 644 (52.2%) |
| t-test | 492 (36.9%) | 305 (62.0%) |  | 570 (36.1%) | 253 (44.4%) |
| Contingency tables | 319 (23.9%) | 154 (48.3%) |  | 523 (33.1%) | 169 (32.3%) |
| Rank transformation nonparametric test | 67 (5.0%) | 29 (43.3%) |  | 187 (11.9%) | 33 (17.7%) |
| ANOVA | 202 (15.1%) | 128 (63.4%) |  | 446 (28.3%) | 263 (59.0%) |
| Repeated-measures analysis | 1 (0.1%) | 1 (100.0%) |  | 17 (1.1%) | 5 (29.4%) |
| Analysis of covariance | 3 (0.2%) | 0 (0.0%) |  | 11 (0.7%) | 1 (9.1%) |
| Pearson’s correlation | 157 (11.8%) | 22 (14.0%) |  | 112 (7.1%) | 5 (4.5%) |
| Simple linear regression | 34 (2.6%) | 5 (14.7%) |  | 19 (1.2%) | 4 (21.1%) |
| Nonparametric correlation | 10 (0.8%) | 0 (0.0%) |  | 44 (2.8%) | 4 (9.1%) |
| Multiple linear regression | 25 (1.9%) | 3 (12.0%) |  | 20 (1.3%) | 1 (5.0%) |
| Logistic regression | 10 (0.8%) | 2 (20.0%) |  | 105 (6.7%) | 8 (7.6%) |
| Survival analysis | 45 (3.4%) | 12 (26.7%) |  | 139 (8.8%) | 6 (4.3%) |
| ROC | 2 (0.2%) | 1 (50.0%) |  | 22 (1.4%) | 3 (13.6%) |
| Others** | 2 (0.2%) | 0 (0.0%) |  | 15 (1.0%) | 0 (0.0%) |

N=total articles (1998: 1335; 2008: 1578)

All articles n (%): percentage=n/N(total articles)×100%,

Incorrect use n (%): n is the number of articles using statistical methods incorrectly; percentage=n/the number of papers using certain statistical methods×100%.

*Including articles do not need statistical methods and articles need statistical methods but omitted (1998: 107; 2008: 76)

**These include nonlinear regression (3 articles), clustering analysis (4), discriminant analysis (1), meta analysis (6), Reliability and validity analysis (3).
