# Supplementary material for: A Retrospective Survey of Research Design and Statistical Analyses in Selected Chinese Medical Journals in 1998 and 2008
Source: PLoS One. 2010 May 25;5(5):e10822. doi: 10.1371/journal.pone.0010822 (PMC2876024; doi:10.1371/journal.pone.0010822)
Supplement: Table S5 — Inappropriate presentation a/o interpretation of results. Presentation and interpretation of results have been improved, but serious errors/defects persisted. Inappropriate presentation of statistical results was the most common defect seen. (0.04 MB DOC) [file pone.0010822.s006.doc]

| **Table S5. Inappropriate presentation a/o interpretation of results** | | |
| --- | --- | --- |
| Inappropriate presentation a/o interpretation of results | 1998 (N=1019)  # articles, n (%)* | 2008 (N=1309)  # articles, n (%)* |
| Using arbitrary *p* thresholds (like p<0.01) instead of reporting exact p-values | 851 (83.5%) | 807 (61.7%) |
| Reporting p value without test statistics | 762 (74.8%) | 754 (57.6%) |
| Insufficient (or inappropriate) description of methods | 537 (52.7%) | 506 (38.7%) |
| *p* values without confidence intervals | 254 (24.9%) | 223 (17.0%) |
| Unspecified statistical methods | 190 (18.7%) | 68 (5.2%) |
| Using mean±SD to describe non-normal data | 28 (2.8%) | 34 (2.6%) |
| Using proportion instead of rate | 0 (0.0%) | 1 (0.1%) |
| Incorrect use of table | 37 (3.6%) | 30 (2.3%) |
| Incorrect selection of chart/figure | 6 (0.6%) | 3 (0.2%) |
| Incorrect use of chart/figure | 22 (2.2%) | 21 (1.6%) |
| Ignoring baselines of two groups | 49 (4.8%) | 20 (1.5%) |
| p<$\alpha$，the smaller the *p* value is, the greater the difference between groups is | 34 (3.3%) | 2 (0.2%) |
| p>$\alpha$，reporting no difference between groups | 4 (0.4%) | 0 (0.0%) |
| p>$\alpha$，reporting difference between groups | 29 (2.9%) | 27 (2.1%) |
| Total for results presentation | 945 (92.7%) | 1023 (78.2%) |
| Total for results interpretation | 99 (9.7%) | 56 (4.3%) |

N=articles need statistical methods=articles used statistical analyses + articles need statistical methods but omitted (1998: 1019=912+107; 2008: 1309=1233+76)

*n (%): percentage=n/N(articles need statistical methods)×100%
